# HDAC6 Inhibitors Rescued the Defective Axonal Mitochondrial Movement in Motor Neurons Derived from the Induced Pluripotent Stem Cells of Peripheral Neuropathy Patients with* HSPB1* Mutation

**DOI:** 10.1155/2016/9475981

**Published:** 2016-12-26

**Authors:** Ji-Yon Kim, So-Youn Woo, Young Bin Hong, Heesun Choi, Jisoo Kim, Hyunjung Choi, Inhee Mook-Jung, Nina Ha, Jangbeen Kyung, Soo Kyung Koo, Sung-Chul Jung, Byung-Ok Choi

**Affiliations:** ^1^Department of Microbiology, School of Medicine, Ewha Womans University, Seoul, Republic of Korea; ^2^Stem Cell & Regenerative Medicine Institute, Samsung Medical Center, Seoul, Republic of Korea; ^3^Department of Biochemistry and Biomedical Sciences, College of Medicine, Seoul National University, Seoul, Republic of Korea; ^4^Chong Kun Dang Research Institute, Yongin-si, Gyeonggi-do, Republic of Korea; ^5^Division of Intractable Diseases, Center for Biomedical Sciences, National Institute of Health, Chungcheongbuk-do, Republic of Korea; ^6^Department of Biochemistry, School of Medicine, Ewha Womans University, Seoul, Republic of Korea; ^7^Departments of Neurology, Samsung Medical Center, Sungkyunkwan University School of Medicine, Seoul, Republic of Korea

## Abstract

The Charcot-Marie-Tooth disease 2F (CMT2F) and distal hereditary motor neuropathy 2B (dHMN2B) are caused by autosomal dominantly inherited mutations of the heat shock 27 kDa protein 1 (*HSPB1*) gene and there are no specific therapies available yet. Here, we assessed the potential therapeutic effect of HDAC6 inhibitors on peripheral neuropathy with* HSPB1* mutation using in vitro model of motor neurons derived from induced pluripotent stem cells (iPSCs) of CMT2F and dHMN2B patients. The absolute velocity of mitochondrial movements and the percentage of moving mitochondria in axons were lower both in CMT2F-motor neurons and in dHMN2B-motor neurons than those in controls, and the severity of the defective mitochondrial movement was different between the two disease models. CMT2F-motor neurons and dHMN2B-motor neurons also showed reduced *α*-tubulin acetylation compared with controls. The newly developed HDAC6 inhibitors, CHEMICAL X4 and CHEMICAL X9, increased acetylation of *α*-tubulin and reversed axonal movement defects of mitochondria in CMT2F-motor neurons and dHMN2B-motor neurons. Our results suggest that the neurons derived from patient-specific iPSCs can be used in drug screening including HDAC6 inhibitors targeting peripheral neuropathy.

## 1. Introduction

Charcot-Marie-Tooth disease (CMT) and related neuropathies are a heterogeneous group of neurodegenerative disorders that share a similar phenotype. So far, almost 40 genes have been identified as being responsible for these disorders [1]. CMT is mainly divided into demyelinating neuropathy (CMT1) and axonal neuropathy (CMT2) according to electrophysiological and histopathological features. Within these types, CMT type 2F (CMT2F) and distal hereditary motor neuropathy 2B (dHMN2B) are caused by heat shock 27 kDa protein 1 (*HSPB1*, also known as* HSP27*) mutation in chromosome 7q11.23 [2]. The most frequent pathologies observed in CMT2F and dHMN2B are abnormal axonal transport and cytoskeleton organization [3–5]. However, our knowledge of the underlying molecular mechanisms is still limited, and specific therapies are not yet available.

HSPB1 belongs to a family of small heat shock proteins (sHSPs) that form dynamic quaternary structures in response to cellular stress via its conserved *α*-crystallin domain. In addition to its role in protein refolding, HSPB1 is involved in protein translation, intracellular reduction/oxidation state, cytoskeletal structure, cell differentiation, and apoptosis [6]. Although HSPB1 is ubiquitously expressed in various tissues, mutations in* HSPB1* appear to cause neuronal degeneration only in the peripheral nervous system, primarily via alteration of cytoskeletal components. Clinically, HSPB1^P182L^ is causative of dHMN2B, whereas HSPB1^S135F^ is causative of both CMT2F and dHMN2B [2]. Previous studies of transfected cell lines indicate that HSPB1^S135F^ expression disrupts the neurofilament (NF) network and increases toxic aggregation of NFs [3], whereas both HSPB1^S135F^ and HSPB1^P182L^ expressions disturb the anterograde transportation of NFs by reducing the binding of kinesin to NFs and inducing cyclin-dependent kinase 5-mediated hyperphosphorylation of NFs [5]. In addition, mutations in* HSPB1* also appear to affect axonal microtubule tracks. In stabilized cell lines and presymptomatic transgenic mice, HSPB1^S135F^ expression leads to aberrant stabilization of microtubulin tracks resulting from hyperactive interaction between HSPB1^S135F^ and *α*-tubulin [4]. Moreover, symptomatic transgenic HSPB1^S135F^ and HSPB1^P182L^ mice show reduced acetylation of *α*-tubulin in sciatic nerves, and HSPB1^S135F^ expressing mice show fewer moving mitochondria in the dorsal root ganglion [7]. However, more physiologically relevant and specific cell models for CMT2F are needed to identify precise disease mechanisms and assess the efficacy of candidate drug screening.

Histone deacetylase 6 (HDAC6) is a class IIb HDAC that regulates acetylation states of *α*-tubulin [8, 9]. Moreover, HDAC6 inhibitors reverse axonal transport deficits in mouse models of CMT2F [7] and cellular models of Parkinson's disease [10] and Huntington's disease [11] by increasing acetylated *α*-tubulin. Therefore, we assessed the therapeutic effect of HDAC6 inhibitors including tubastatin A and newly developed CHEMICAL X4 and CHEMICAL X9 (Chong Kun Dang Pharmaceutical Cooperation, Seoul, Korea), in CMT2F- and dHMN2B-specific motor neurons (MNs) differentiated from induced pluripotent stem cells (iPSCs) from patients carrying HSPB1^S135F^ and HSPB1^P182L^ mutations, by analyzing mitochondrial movements and acetylation of *α*-tubulin in vitro.

## 2. Materials and Methods

### 2.1. Ethics Statement and Human Samples

Skin fibroblasts were obtained from skin biopsy (4 mm punch) of two patients carrying mutant forms of HSPB1 (S135F and P182L) with informed consent after Institutional Review Board approval (ECT 11-58-37, Ewha Womans University, Mok-Dong Hospital, Seoul, Korea). All experiments were performed in accordance with the approved guidelines and regulations. Fresh skin samples were minced and digested in Dulbecco's Modified Eagle's Medium (DMEM) containing 10 mg/mL collagenase type IV, 50 U/mL dispase, and 0.05% trypsin/EDTA for 40 min at 37°C. After filtration through a cell strainer (70 *μ*m pore size), cells were washed twice and harvested in DMEM containing 20% fetal bovine serum (FBS) and 100 *μ*g/mL penicillin/streptomycin.

### 2.2. Generation of iPSCs

Four types of Sendai virus-containing vectors, each expressing different transcription factors (*Sox2*,* Oct4*,* Klf4*, and* c-Myc*; Invitrogen, Carlsbad, CA, USA), were introduced into fibroblasts derived from patients with a multiplicity of infection of 3 by following manufacturer's protocol. On day 7, cells were trypsinized and transferred onto mitomycin C (Sigma, St. Louis, MO, USA)-treated mouse embryonic fibroblasts, SNL feeder cells (Cell Biolabs, Inc., San Diego, CA, USA), and harvested with embryonic stem cell (ESC)/iPSC medium (KnockOut, Gibco, Grand Island, NY, USA) containing 4 ng/mL basic fibroblast growth factor (bFGF). The medium was changed daily. On day 30, iPSC colonies were selected based on their morphological characteristics. Other stem cells used as controls included human ESCs (WA09; WiCell, Madison, WI, USA) and human iPSCs (hFSiPS1; National Stem Cell Bank of Korea, Cheongju, Korea).

### 2.3. Karyotype Analysis

CMT2F-iPSCs and dHMN2B-iPSCs were cultured without feeder cells at cellular passages of 10, and 20 cells from each type of iPSCs were analyzed for the karyoptype by Seegene Medical Foundation (Seoul, Korea).

### 2.4. Teratoma Assay

Equal volumes of mixtures of Matrigel (Corning, Corning, NY, USA) and 1.0 × 10^6^ human ESCs (WA09), human iPSCs (hFSiPS1), CMT2F-iPSCs, or dHMN2B-iPSCs were subcutaneously injected into the backs of 5-week-old female NOD/SCID mice (Laboratory Animal Resource Center, Korean Research Institute of Bioscience and Biotechnology, Daejeon, Korea). The xenografts were allowed to grow for 8 weeks and were then explanted by surgical procedures. Teratoma tissues were fixed in 10% formaldehyde and embedded in paraffin. For histological analysis, hematoxylin and eosin staining was performed.

### 2.5. Embryoid Body- (EB-) Mediated In Vitro Differentiation Assay

ESCs and iPSCs were plated onto ultralow binding plates (i.e., uncoated Petri dishes) and cultured in suspension with ESC/iPSC medium (KnockOut), which was replaced every other day. After 8 days in floating culture, EBs were transferred onto a gelatin-coated (Sigma) chamber slide (Nalgene/Nunc, Rochester, NY, USA) and cultured in 10% FBS/DMEM (Welgene, Daegu, Korea) to allow differentiation randomly into three germ-layered cells for an additional 8 days.

### 2.6. Reverse Transcription Polymerase Chain Reaction (RT-PCR)

For the detection of total and endogenous expression of* KLF4, OCT4, SOX2,* and c*-MYC* and* HSPB1*, RNA extraction from ESCs and iPSCs was performed with TRIzol. Reverse transcription was performed using AMV reverse transcriptase (Promega, Madison, WI, USA), and PCR was performed using* Ex Taq* polymerase (Takara Bio, Otsu, Japan). Primer sequences are KLF4 CDR (108 bp) 5′-CTG CGG CAA AAC CTA CAC AAA-3′ (forward) and 5′-GCG AAT TTC CAT CCA CAG CC -3′ (reverse); KLF4 UTR (96 bp) 5′-CAT GGT CAA GTT CCC AAC TGA G-3′ (forward) and 5′-CAC AGA CCC CAT CTG TTC TTT G-3′ (reverse); OCT3/4 CDR (161 bp) 5′-CAG TGC CCG AAA CCC ACA C-3′ (forward) and 5′-GGA GAC CCA GCA GCC TCA AA-3′ (reverse); OCT3/4 UTR (120 bp) 5′-GAA AAC CTG GAG TTT GTG CCA-3′ (forward) and 5′-TCA CCT TCC CTC CAA CCA GTT-3′ (reverse); SOX2 CDR (131 bp) 5′-TAC CTC TTC CTC CCA CTC C-3′ (forward) and 5′-GGT AGT GCT GGG ACA TGT GA-3′ (reverse); SOX2 UTR (105 bp) 5′-CCC GGT ACG CTC AAA AAG AA-3′ (forward) and 5′-GGT TTT TGC GTG AGT GTG GAT-3′ (reverse); c-MYC CDR (380 bp) 5′-CGT CCT CGG ATT CTC TGC TC-3′ (forward) and 5′-GCT GGT GCA TTT TCG GTT GT-3′ (reverse); c-MYC UTR (328 bp) 5′-GCG TCC TGG GAA GGG AGA TCC GGA GC-3′ (forward) and 5′-TTG AGG GGC ATC GTC GCG GGA GGC TG-3′ (reverse).

### 2.7. Sanger Sequencing

Pathogenic mutations (404C>T and 545C>T) in HSPB1 gene from patients iPSCs were confirmed by Sanger sequencing using a 3730xl DNA Analyzer (Macrogen Inc., Seoul, Korea) and analyzed using Sequencher v.5.2.3 (GeneCodes Corporation, Ann Arbor, MI, USA). The primers used for amplifying and sequencing are as follows: 5′-TTT CTG AGC AGA CGT CCA GA-3′ (forward) and 5′-CTT TAC TTG GCG GCA GTC TC-3′ (reverse).

### 2.8. Directed Differentiation of iPSCs into MNs

To generate EBs, colonies of ESCs and iPSCs were enzymatically dissociated into small clumps and cultured in suspension for 2 days in a Petri dish supplemented with ESC/iPSC medium (KnockOut) containing 10 *μ*M Rho-associated kinase inhibitor Y27632 (Tocris Bioscience, Bristol, UK), 20 ng/mL bFGF (Invitrogen), 10 *μ*M SB435142 (Stemgent, Cambridge, MA), and 0.2 *μ*M LDN193189 (Stemgent) and penicillin/streptomycin. On day 5, for caudalization, retinoic acid (1 *μ*M; Sigma), ascorbic acid (0.4 *μ*g/mL; Sigma), brain-derived neurotrophic factor (10 ng/mL; R&D, Minneapolis, MN, USA), and N2 supplement (1%; Gibco) were added [12]. On day 7, for ventralization, the sonic hedgehog agonist purmorphamine (1 *μ*M; Stemgent) was added, and the dual SMAD inhibitors (SB435142 and LDN193189) were discontinued. On day 17, basal medium was changed to neurobasal medium (Invitrogen) containing all previously indicated factors with the addition of insulin-like growth factor-1 (10 ng/mL), glial cell line-derived neurotrophic factor (10 ng/mL), and B27 (2%; Gibco). On day 21, neurospheres were dissociated with accutase (Gibco) and plated onto poly-L-lysine/laminin-coated culture plates or slide chambers (Nalgene) and supplemented with neurobasal medium containing all previously indicated factors with the addition of *β*-mercaptoethanol (25 *μ*M; Gibco) and glutamic acid (25 *μ*M; Sigma).

### 2.9. Immunoblot Assay

Proteins were collected from differentiated MNs by conventional methods using RIPA lysis buffer (150 mM NaCl, 1.0% NP-40, 0.5% sodium deoxycholate, 0.1% sodium dodecyl sulfate, 50 mM Tris, pH 8.0). SDS-PAGE gel was transferred to polyvinylidene difluoride membranes. For blotting, anti-acetylated *α*-tubulin (1 : 1000; mouse IgG_2b_, 6-11B-1, Abcam, Cambridge, UK) and anti-*α*-tubulin (1 : 1000; mouse IgG_1_, DM1A, Sigma) antibodies were used. Band densities were analyzed with UN-SCAN-IT gel software (Silk Scientific, Inc., Orem, UT, USA).

### 2.10. Immunocytochemistry

Cells were fixed with 4% paraformaldehyde and blocked with 10% normal goat serum (Gibco) and 0.2% Triton X-100. Primary antibodies were anti-SSEA4 (1 : 100; mouse IgG_3_, MC-813-70, Developmental Studies Hybridoma Bank (DSHB), Iowa City, IA, USA), anti-NANOG (1 : 500; mouse IgG_1_, NNG-811, Abcam), anti-*α*-fetoprotein (AFP; 1 : 100; mouse IgG_2b_, 2A9, Abcam), anti-*α*-smooth muscle actin (SMA; 1 : 100; mouse IgG_2a_, 1A4, Abcam), anti-nestin (1 : 1000; mouse IgG1, 10C2, Abcam), anti-HB9 (1 : 100; mouse IgG1, 81.5C10, DSHB), anti-islet-1/2 (ISL1/2; 1 : 50; mouse IgG_2b_, 39.4DS, DSHB), anti-neurofilament H nonphosphorylated (SMI32; 1 : 500; mouse IgG_1_, Covance, Princeton, NJ, USA), anti-neuron-specific beta III tubulin (Tuj1; 1 : 1000; rabbit polyclonal, Abcam), anti-microtubule-associated protein 2 (MAP2; 1 : 200; rabbit polyclonal, Millipore, Billerica, MA, USA), anti-choline acetyltransferase (ChAT; 1 : 1000; rabbit polyclonal, Abcam), anti-synapsin 1 (1 : 1000; rabbit polyclonal, Abcam), anti-*α*-tubulin (1 : 500; rabbit polyclonal, Abcam), and anti-acetylated *α*-tubulin (1 : 200; mouse IgG_2b_, 6-11B-1, Abcam) antibodies. Secondary antibodies were Alexa Fluor 488-conjugated goat anti-rabbit IgG (preadsorbed; Abcam), Cy3-conjugated goat anti-rabbit IgG (Abcam), FITC-conjugated goat anti-mouse IgG (Abcam), and Cy3-conjugated goat anti-mouse IgG (Abcam) antibodies.

### 2.11. Microfluidic Culture for Analysis of Axonal Mitochondrial Movements

Neurospheres derived from ESCs and iPSCs were dissociated with accutase into single cells and seeded onto microchannel plates at a density of 1 × 10^5^ cells/plate and cultured with neurobasal (Invitrogen) and B27 medium for 10 days. After axons had completely stretched through the *μ*m-sized grooves (total length = 833.4 *μ*m) and reached the opposite compartment, MNs were transfected with mito-dsRED2 (Clonetech Inc./Takara Bio) delivered by lipofectamine 2000 (Invitrogen). Within 3 days of transfection, mitochondrial images were captured by a fluorescent microscope at a rate of 121 snaps/2 min and stacked into one file to create kymographs. Mitochondrial moving velocity was calculated by measured angle and distance in kymograph using ImageJ software. Axonal lengths were also measured using ImageJ. Axonal lengths were calculated by summation of measured length of axon stretched out from a single *μ*m-sized groove and known length of the groove (833.4 *μ*m).

### 2.12. Drug Treatment

For analyzing protein acetylation level, cells were treated with the HDAC6 inhibitors: tubastatin A (5 *μ*M; Selleckchem, Houston, TX, USA), CHEMICAL X4 (0.5 *μ*M and 5 *μ*M), and X9 (0.5 *μ*M and 5 *μ*M) for 16 hrs. For analyzing axonal transport, HDAC6 inhibitors mentioned above were treated for 3 hrs. CHEMICAL X4 and X9 are newly established HDAC6 inhibitors from Chong Kun Dang Pharmaceutical Cooperation (Seoul, Korea). The common structures of X4 and X9 are composed of three components (the Zn-binder, the linker and the cap part) and are very potent hydroxamate-based HDAC6 inhibitors [IC_50_ (HDAC6) = 5.3 and 3.7 nM, resp.]. Similar to pan-HDAC or other HDAC subtype specific inhibitors, the hydroxamic acid moiety as a Zn-binding group is very important for blocking the catalytic activity of the HDAC6. Even though these compounds have structurally different cap portions (arylurea and indole), they share the benzyl group as a linker moiety resulting in the sufficient HDAC6-specificity [fold (HDAC1/HDAC6) = 400 and >270, resp.] to afford the desirable efficacies in cells and animals without the toxicities associated with the inhibitions of class I HDAC subtypes.

### 2.13. Statistical Analysis

Values are expressed as mean ± standard error of the mean. Groups were compared using two-way analysis of variance using GraphPad Prism (version 5; GraphPad Software Inc., La Jolla, CA, USA). Statistical significance was set at *P* < 0.05.

## 3. Results

### 3.1. Generation of CMT2F-iPSCs and dHMN2B-iPSCs

Patient-specific iPSCs were generated from one CMT2F patient (female/52-year-old, Korean) with 404C>T (S135F) mutation and one dHMN2B patient (female/8-year-old, Korean) with 545C>T (P182L) mutation of the* HSPB1 *gene. Both of CMT2F and dHMN2B patients showed predominant distal leg muscle weakness and toe gait abnormalities. The ages at onset were 20 years and 7 years, respectively. Sensory neve conduction velocities and action potentials of sural nerves were decreased in CMT2F patient, but within normal ranges in dHMN2B patient.

**Figure 1:**
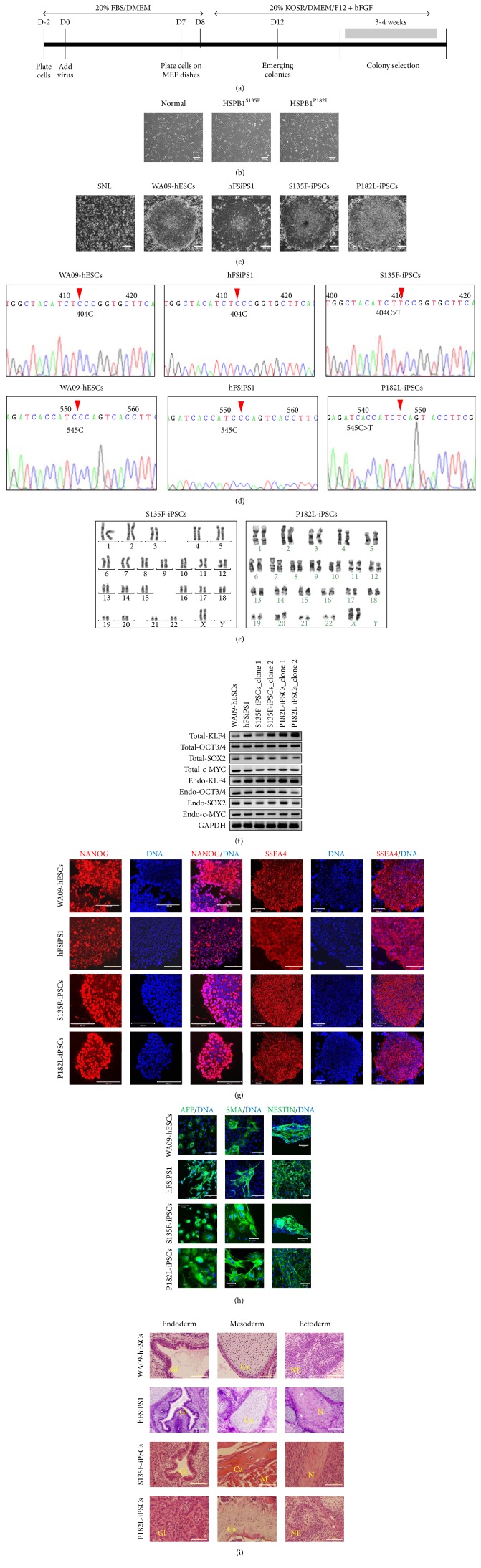
Generation of CMT2F patient and dHMN2B patient-derived iPSCs. (a) Experimental timeline for iPSC generation. KOSR, KnockOut™ serum replacement; bFGF, basic fibroblast growth factor. (b) Morphology of fibroblasts from normal individual and patients (original magnification, 50x). Scale bars: 200 *μ*m. (c) iPSC colonies showed ESC-like morphology, such as a flat cobblestone-like appearance with individual cells having a high nucleus-to-cytoplasm ratio (original magnification, 50x). Scale bars: 200 *μ*m. (d) CMT2F-iPSCs and dHMN2B-iPSCs had preserved point mutation sites in the* HSPB1* gene, verified by sequencing of RT-PCR products. (e) CMT2F-iPSCs and dHMN2B-iPSCs maintained normal karyotype. (f) Expression of total and endogenous* Klf4, Oct3/4, Sox2*, and* c-Myc* in CMT2F-iPSCs and dHMN2B-iPSCs was verified by RT-PCR. Two clones from each of the patients-derived iPSCs were tested (clone 1 and clone 2). (g) ESCs and iPSCs expressed stem cell markers such as NANOG (in the nucleus; original magnification, 200x) and SSEA4 (in the cytoplasm; original magnification, 100x). Scale bars: 200 *μ*m. (h) EB-mediated in vitro spontaneous differentiation of ESCs and iPSCs resulted in the expression of three-germ-layer markers such as AFP (endoderm), SMA (mesoderm), and nestin (ectoderm; original magnification, 200x). Scale bars: 100 *μ*m. (i) ESCs and iPSCs showed in vivo pluripotency by forming teratomas 8 weeks after subcutaneous injection into NOD/SCID mice. Teratomas consisted of various three-germ-layer tissues including columnar gland epithelial cells with secretions [Gl] for endodermal tissue, muscle [M] and cartilage with calcification [Ca] for mesodermal tissue, and neuroectodermal tissue [NE] and nerve axonal bundle [N] for ectodermal tissue (original magnification, 400x). Scale bars: 100 *μ*m.

Skin fibroblasts were reprogrammed into iPSCs by Sendai viral transduction of four episomal vectors carrying* KLF4, OCT3/4, SOX2,* and* c-MYC *(Figures 1(a) and 1(b)). The morphology of CMT2F-iPSC and dHMN2B-iPSC colonies, which resembled that of human ESCs (WA09), consisted of cells having a high nuclear-to-cytoplasm ratio compacted in a flat cobblestone-like appearance with sharp edges (Figure 1(c)). The genetic background of CMT2F-iPSCs and dHMN2B-iPSCs was not changed during the reprogramming processes, especially at the mutation site of* HSPB1* (Figure 1(d)). CMT2F-iPSCs and dHMN2B-iPSCs preserved their normal karyotype (Figure 1(e)). The expression of endogenous* KLF4, OCT3/4, SOX2,* and* c-MYC *genes was detected by RT-PCR after a few subpassages using primers with complementary sequences to the intron area of the target mRNA (Figure 1(f)). Sendai viral genome contents were not detected in iPSCs after cellular passage of 10 (see Supplement Figure S1 in Supplementary Material available online at http://dx.doi.org/10.1155/2016/9475981). CMT2F-iPSCs and dHMN2B-iPSCs expressed stem cell markers such as NANOG in the nucleus and SSEA in the cytoplasm (Figure 1(g)). The pluripotency of CMT2F-iPSCs and dHMN2B-iPSCs was verified by the presence of randomly differentiated AFP-positive endodermal cells, SMA-positive mesodermal cells, and nestin-positive ectodermal cells via EB formation in vitro (Figure 1(h)) and teratoma formation in vivo (Figure 1(i)).

### 3.2. Derivation of In Vitro Models by MN Differentiation of CMT2F-iPSCs and dHMN2B-iPSCs

**Figure 2:**
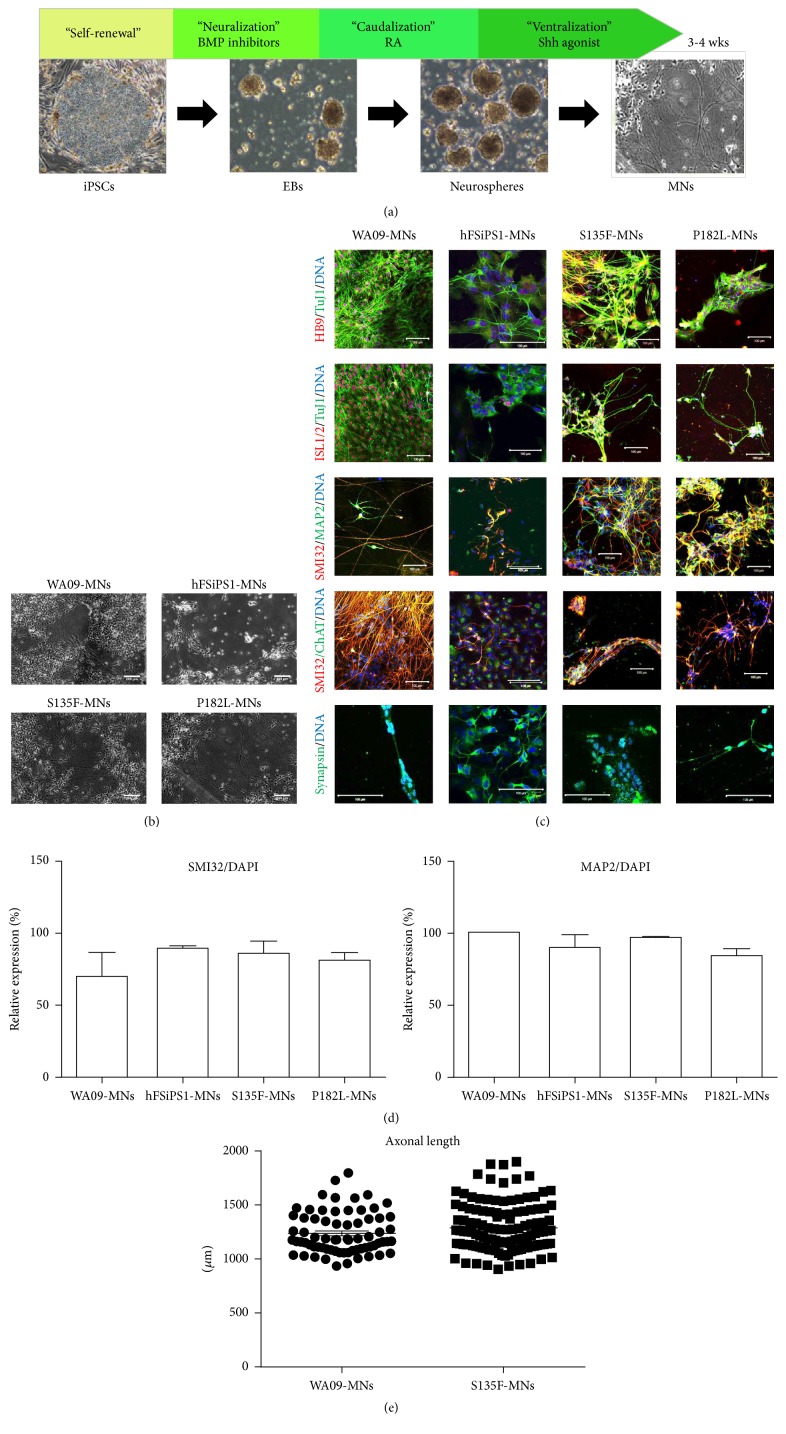
Generation of CMT2F-specific in vitro model by differentiation of patient-derived iPSCs into MNs. (a) Schematic presentation of MN differentiation. (b) Differentiated MNs showed typical cellular morphology (original magnification, 50x). Scale bars: 200 *μ*m. (c) S135F-MNs and P182L-MNs expressed MN-specific transcription factors such as HB9 and ISL1/2; neuronal cytoskeletal markers such as TuJ1, SMI32, and MAP2; ChAT; and the synaptic vesicular marker synapsin (original magnification, 400x and 600x). Scale bars: 100 *μ*m. (d) Differentiation efficiency of S135F-MNs and P182L-MNs was comparable to control MNs in terms of neuronal marker expression such as SMI32/DAPI and MAP2/DAPI (WA09-MNs; *N* = 30, hFSiPS1-MNs; *N* = 329, S135F-MNs; *N* = 1730, and P182L-MNs; *N* = 1090). (e) Axonal length of S135F-MNs was comparable to that of control MNs. Axonal length was measured by culturing fully differentiated MNs in microchannel plates for an additional 2 weeks (WA09-MNs: *N* = 70 and S135F-MNs: *N* = 121).

To recapitulate peripheral neuropathy, MNs were differentiated from iPSCs by providing dual SMAD inhibitors (SB435142 and LDN193189) for neuralization, retinoic acid for caudalization, and purmorphamine for ventralization according to the method described by Amoroso et al. [12] (Figure 2(a)). Fully differentiated MNs (Figure 2(b)) expressed transcription factors such as HB9 and ISL1/2, cytoskeletal markers such as Tuj1, MAP2, and SMI32, and synapsin and ChAT (Figure 2(c)). S135F-MNs and P182L-MNs showed no developmental defects, evidenced by no differences between S135F-MNs and P182L-MNs and control WA09-MNs and hFSiPS1-MNs in the proportion of marker-positive cells (Figure 2(d)). WA09-MNs (1236 ± 23 *μ*m) and S135F-MNs (1287 ± 20 *μ*m) showed no differences in axonal length (Figure 2(e)). Neuromuscular junctions, visualized by *α*-bungarotoxin staining (see Supplement Figure S2), formed when MNs were cocultured with myotube cells differentiated from C2C12 cells.

### 3.3. Axonal Mitochondrial Transport Defects in S135F-MNs

**Figure 3:**
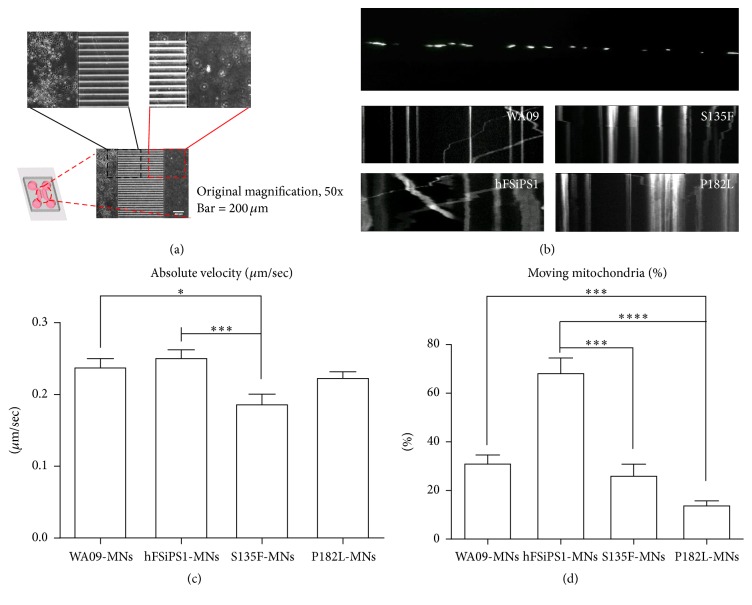
Axonal transport of mitochondria in control MNs, S135F-MNs and P182L-MNs. (a) Microfluidic culturing system. Microchannel plates were made from transparent polymer in a specialized mold to create multicompartments connected by *μ*m-sized grooves (833.4 *μ*m long in length) that only allow growing axons to pass through, thereby separating the microenvironments of neuronal components. (b) Visualization of mitochondria by Mito-dsRED2 transfection (upper image) and kymograph images for mitochondria movement (middle and lower four images). Mito-dsRED2 vector was introduced into fully differentiated MNs cultured on microchannel plates for an additional 1 week. Mitochondrial images were taken at a ratio of 121 snaps/2 min and stacked into kymograph for analysis (original magnification, 1000x). (c) S135F-MNs show decreased velocity of moving mitochondria compared to control MNs. Unpaired* t*-tests,^*∗*^ *P* < 0.05, ^*∗∗∗*^ *P* < 0.001, and ^*∗∗∗∗*^ *P* < 0.0001. Absolute velocity of moving mitochondria was calculated from measured angle and length in kymograph images using ImageJ (WA09-MNs; *N* = 203, hFSiPS1-MNs; *N* = 512, S135F-MNs; *N* = 428, and P182L-MNs; *N* = 496). (d) S135F-MNs and P182L-MNs show reduced moving proportion of mitochondria in an axon (WA09-MNs; *N* = 9, hFSiPS1-MNs; *N* = 6, S135F-MNs; *N* = 20, and P182L-MNs; *N* = 42).

Although there is heterogeneity in causative genes for different CMT2 subtypes, many disease subtypes involve abnormalities in the cellular trafficking system [13]. As MNs can have long axons up to one meter in length, defects in axonal transportation may increase vulnerability to axonopathy. In particular, mitochondrial transport is extremely important for maintaining axonal and synaptic stability in neurons. During bidirectional trafficking of mitochondria along microtubules, quality control is accomplished by dynamic fusion and fission processes that enable mitochondria to generate ATP to support vital cellular functions and buffer intracellular calcium [14]. Therefore, we tested whether S135F-MNs and P182L-MNs have defects in mitochondrial axonal transport by culturing cells in microchannel plates [15], which compartmentalize axons from soma and dendrites (Figure 3(a)), transfecting cells with mito-dsRED2, and analyzing kymograph images (Figure 3(b)). We observed that the absolute velocity of mitochondrial movements was significantly lower in S135F-MNs (0.19 ± 0.01 *μ*m/sec) and slightly lower in P182L-MNs (0.22 ± 0.01 *μ*m/sec) compared with control WA09-MNs (0.24 ± 0.01 *μ*m/sec) and hFSiPS1-MNs (0.25 ± 0.01 *μ*m/sec) (Figure 3(c), Supplement Figure S3, and Supplement Table S1). Also the proportion of moving mitochondria was significantly decreased in S135F-MNs (26.37 ± 5.06%) and P182L-MNs (14.19 ± 2.14%) compared to those in control WA09-MNs (31.39 ± 3.74%) and hFSiPS-MNs (Figure 3(d) and Supplement Table S2).

### 3.4. Decreased Acetylation of *α*-Tubulin in S135F-MNs

**Figure 4:**
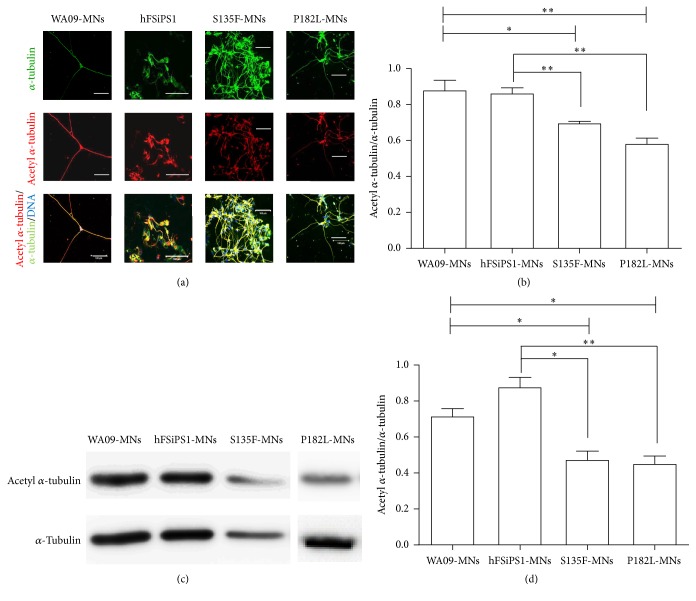
Reduced acetylation of *α*-tubulin in S135F-MNs and P182-MNs. (a) Acetylation of *α*-tubulin was visualized by immunostaining with anti-acetylated *α*-tubulin Abs (red). Total *α*-tubulin was also detected by staining with anti-*α*-tubulin Abs (green) (original magnification, 400x). Scale bars: 100 *μ*m. (b) The extent of acetylated *α*-tubulin was normalized with corresponding total *α*-tubulin using ImageJ software (WA09-MNs; *N* = 3, S135F-MNs; *N* = 4, and P182L-MNs; *N* = 5). Unpaired *t*-tests, ^*∗*^ *P* < 0.05 and ^*∗∗*^ *P* < 0.01. (c and d) Western blot assay showed decreased acetylated *α*-tubulin levels in S135F-MNs and P182-MNs (paired *t*-tests, ^*∗*^ *P* < 0.05 and ^*∗∗*^ *P* < 0.01).

Axonal transport is regulated by various posttranslational modifications (e.g., detyrosylation, acetylation, and glutamylation) of microtubules through the recruitment of molecular motor proteins [16]. Among these modifications, acetylation of *α*-tubulin at the protein site of K40 regulates binding of dynein/dynactin complexes and kinesin-1 to microtubules [11, 17]. In addition, treatment with HDAC6 inhibitors, which increase acetylation of *α*-tubulin, reverses transport defects in cellular models of Huntington's disease [11] and LRRK2 mutation-induced Parkinson's disease [9]. Therefore, acetylation levels of *α*-tubulin were examined as an indicator of mitochondrial transport defects in S135F-MNs and P182L-MNs. We found that S135F-MNs and P182L-MNs showed a significant reduction in acetylated *α*-tubulin levels compared with WA09-MNs or hFSiPS1-MNs (Figure 4).

### 3.5. CMT2F- and dHMN2B-Related Disease Phenotypes Were Reversed by HDAC6 Inhibition

**Figure 5:**
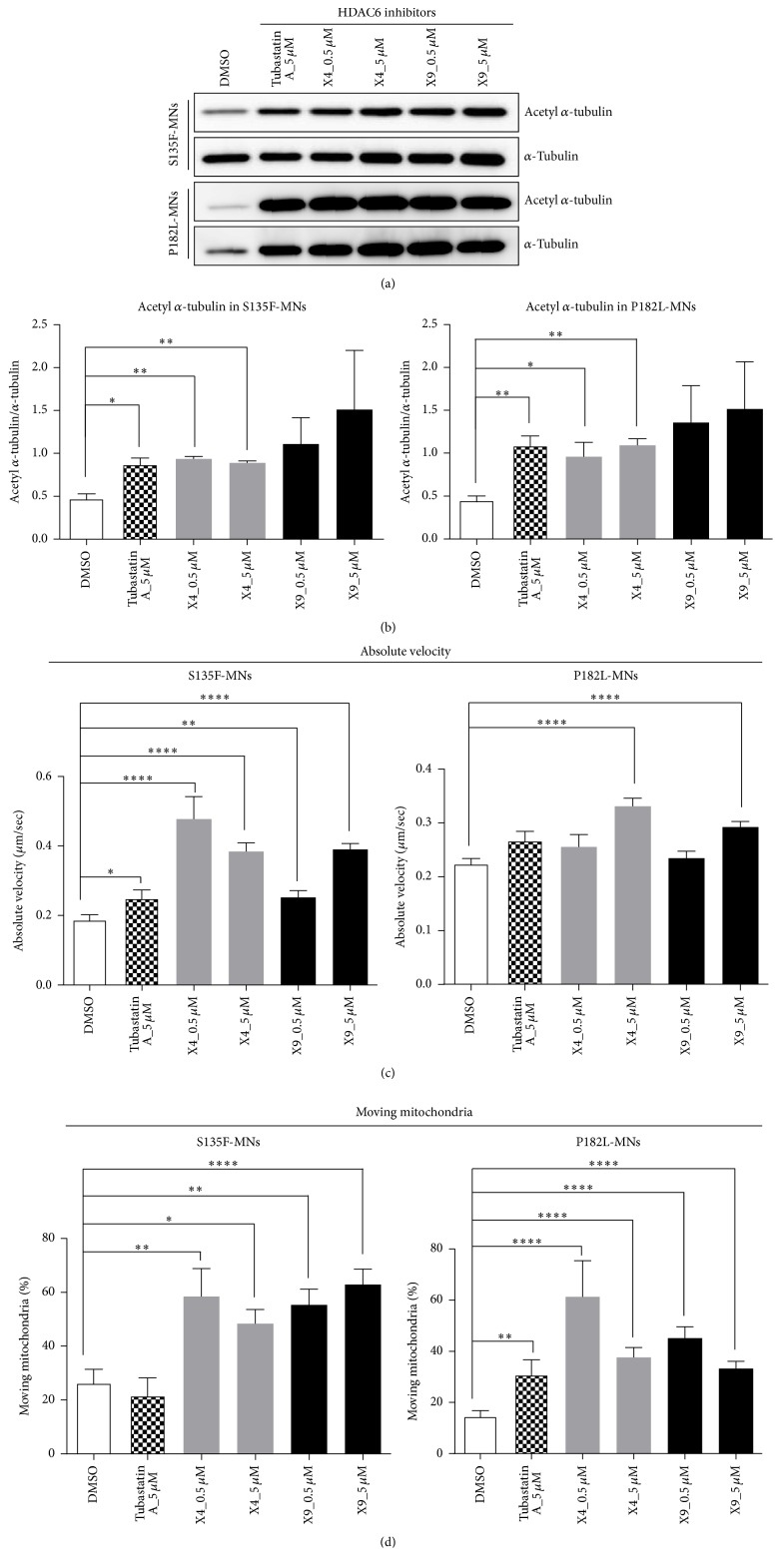
HDAC6 inhibitors reversed axonal defect of mitochondrial transport by increasing acetylation of *α*-tubulin. (a and b) Western blot assay showed that treatment of HDAC6 inhibitors increased acetylated *α*-tubulin levels in S135F-MNs and P182L-MNs (paired *t*-tests, ^*∗*^ *P* < 0.05 and ^*∗∗*^ *P* < 0.01). (c) HDAC6 inhibitors increased the absolute velocity of mitochondrial movements in S135F-MNs and P182L-MNs (WA09-MNs; *N* = 617, S135F-MNs; *N* = 101, and P182L-MNs; *N* = 394). (d) HDAC6 inhibitors increased the percentage of moving mitochondria in S135F-MNs and P182L-MNs (WA09-MNs; *N* = 15, S135F-MNs; *N* = 7, and P182L-MNs; *N* = 15). Unpaired *t*-tests, ^*∗*^ *P* < 0.05, ^*∗∗*^ *P* < 0.01, and ^*∗∗∗∗*^ *P* < 0.0001.

By the treatment with HDAC6 inhibitors, including tubastatin A (5 *μ*M) and newly developed CHEMICAL X4 (0.5 *μ*M and 5 *μ*M) and X9 (0.5 *μ*M and 5 *μ*M), acetylation of *α*-tubulin in S135F-MNs and P182L-MNs was increased (Supplement Figure S4, Figures 5(a) and 5(b)). Moreover, the absolute velocity of mitochondrial movements and the proportion of moving mitochondria in axons were increased significantly by the treatment of these HDAC6 inhibitors (Figures 5(c) and 5(d) and Supplement Tables S1 and S2).

## 4. Discussion

Since iPSCs serve as an unlimited source of cells that can give rise to neuronal lineages and help to overcome the obstacle of unavailability of affected neuronal tissues from patients due to neuronal postmitotic property, in vitro modeling and drug screening with iPSCs can advance basic research on neurodegenerative diseases. Therefore, one of the strongest points of this study is that CMT2F- and dHMN2B-specific in vitro cellular models can be applied for the screening of putative therapeutic molecules, such as HDAC6 inhibitors on peripheral neuropathy.

HDAC6 is a type IIb HDAC that modulates deacetylation of cytoplasmic target proteins. Because HDAC6 is involved in cytoskeletal formation, modulation of cellular oxidation/reduction state, and the ubiquitin-proteasome system, inhibition of HDAC6 improves pathological conditions in neurons such as impairments in axonal transport, oxidative stress, and protein aggresome formation in vitro [18, 19]. In terms of axonal transport, HDAC6 inhibitors reverse CMT2F and dHMN2B-related axonal transport by increasing acetylated *α*-tubulin. In this study, newly developed HDAC6 inhibitors having 3.5-fold lower half-maximal inhibitory concentration (IC_50_) values than that of tubastatin A showed better performance on improving mitochondrial axonal transport at 10 times lower concentration than tubastatin A.

CMT2F-iPSCs and dHMN2B-iPSCs were generated from patient-derived dermal fibroblasts carrying 404C>T (S135F) and 545C>T (P182L) mutations of HSPB1 by forced expression of* KLF4, OCT3/4, SOX2,* and* c-MYC* using Sendai virus vectors. After establishing several stem cell colonies, two colony clones from each patient were used for further analysis to avoid differences due to clonal variance and selected iPSCs were differentiated into motor neurons with defined cytokines. To recapitulate axonal pathology in vitro, patient-specific motor neurons were cultured in microchanneled plates which are designed to separate the microenvironments of neuronal compartments. In this system, specific cellular models of CMT2F and dHMN2B showed defects in mitochondrial transport, as the absolute velocity of moving mitochondria and the percentage of moving mitochondria were lower than control MNs. Phenotypic variance between iPSC clones was observed in P182L-MNs in terms of mitochondrial velocity (see Figure S3). Defects in the axonal traffic system are linked to many neurodegenerative diseases. Due to the distinct anatomy of peripheral neurons, which have axons up to one meter in length, the interchange of genetic material, metabolites, proteins, and organelles between soma and distal subcellular sites is critical for maintaining neuronal homeostasis and survival. Of the various trafficking cargos, the transport of mitochondria is particularly important because mitochondria support several cellular processes by supplying ATP, buffering intracellular Ca^2+^, and modulating apoptosis [13]. In addition to delivering mitochondria to appropriate subcellular sites, the trafficking of mitochondria is vital for the recycling of their components through dynamic fusion and fission mechanism [14]. Defects in axonal mitochondrial transport are associated with Alzheimer's disease [20, 21], amyotrophic lateral sclerosis [22], and Huntington's disease [23, 24].

Mitochondria are transported bidirectionally by interactions with molecular motor proteins, such as kinesin-1 for anterograde movement and dynein for retrograde movement, and those attach via adaptor proteins such as the mitochondrial Rho-Milton complex and dynactin, respectively [14]. The recruitment of molecular motors to microtubule tracks is regulated by posttranslational modification of *α*-tubulin [16], especially acetylation [17]. In this study, the level of acetylated *α*-tubulin was reduced in both S135F-MNs and P182L-MNs. By increasing the acetylation of *α*-tubulin via treatment with HDAC6 inhibitors, mitochondrial transport was improved in terms of both velocity and the percentage of moving mitochondria. Hence, defects in the axonal trafficking of mitochondria are associated with decreased acetylation of *α*-tubulin, which may be the main causative factor for axonopathy in CMT2F and dHMN2B.

In summary, iPSCs can be used for valuable resources of in vitro disease modeling as well as drug screening. We anticipate that this in vitro system would be a useful model for the clinical translational research and drug development.

## 5. Conclusions

This study demonstrates in vitro drug screening models of inherited neuropathy resulting from mutations in the* HSPB1* gene can be developed from patient-specific iPSCs. Axonal pathologies were mimicked in microfluidic culturing system as both S135F-MNs and P182L-MNs showed a marked decline in the absolute velocity and the percentage of moving mitochondria. These axonal defects were associated with decreased acetylation of *α*-tubulin and were reversed by treatment of HDAC6 inhibitors.

## Supplementary Material

Supplementary Material contains PCR detection of Sendai viral genome in iPSCs, and neuromuscular junction staining. It also contains mitochondrial movement data of different two clones from the patients. For comparison, immunostaining of acetylated alpha-tubulin data in S135F-MNs and P182L-MNs with HDAC6 inhibitors are included.

## References

[1] Inherited Peripheral Neuropathies Mutation Database http://www.molgen.ua.ac.be/cmtmutations/mutations/MutByGene.cfm.

[2] Evgrafov O. V., Mersiyanova I., Irobi J. (2004). Mutant small heat-shock protein 27 causes axonal Charcot-Marie-Tooth disease and distal hereditary motor neuropathy. *Nature Genetics*.

[3] Zhai J., Lin H., Julien J.-P., Schlaepfer W. W. (2007). Disruption of neurofilament network with aggregation of light neurofilament protein: a common pathway leading to motor neuron degeneration due to Charcot-Marie-Tooth disease-linked mutations in NFL and HSPB1. *Human Molecular Genetics*.

[4] Almeida-Souza L., Asselbergh B., d'Ydewalle C. (2011). Small heat-shock protein HSPB1 mutants stabilize microtubules in Charcot-Marie-Tooth neuropathy. *The Journal of Neuroscience*.

[5] Holmgren A., Bouhy D., De Winter V. (2013). Charcot-Marie-Tooth causing HSPB1 mutations increase Cdk5-mediated phosphorylation of neurofilaments. *Acta Neuropathologica*.

[6] Arrigo A.-P. (2007). The cellular “networking” of mammalian Hsp27 and its functions in the control of protein folding, redox state and apoptosis. *Advances in eXperimental Medicine and Biology*.

[7] D'Ydewalle C., Krishnan J., Chiheb D. M. (2011). HDAC6 inhibitors reverse axonal loss in a mouse model of mutant HSPB1-induced Charcot-Marie-Tooth disease. *Nature Medicine*.

[8] Zhang Y., Li N., Caron C. (2003). HDAC-6 interacts with and deacetylates tubulin and microtubules in vivo. *EMBO Journal*.

[9] Valenzuela-Fernández A., Cabrero J. R., Serrador J. M., Sánchez-Madrid F. (2008). HDAC6: a key regulator of cytoskeleton, cell migration and cell-cell interactions. *Trends in Cell Biology*.

[10] Godena V. K., Brookes-Hocking N., Moller A. (2014). Increasing microtubule acetylation rescues axonal transport and locomotor deficits caused by LRRK2 Roc-COR domain mutations. *Nature Communications*.

[11] Dompierre J. P., Godin J. D., Charrin B. C. (2007). Histone deacetylase 6 inhibition compensates for the transport deficit in Huntington's disease by increasing tubulin acetylation. *The Journal of Neuroscience*.

[12] Amoroso M. W., Croft G. F., Williams D. J. (2013). Accelerated high-yield generation of limb-innervating motor neurons from human stem cells. *Journal of Neuroscience*.

[13] Gentil B. J., Cooper L. (2012). Molecular basis of axonal dysfunction and traffic impairments in CMT. *Brain Research Bulletin*.

[14] Sheng Z.-H., Cai Q. (2012). Mitochondrial transport in neurons: impact on synaptic homeostasis and neurodegeneration. *Nature Reviews Neuroscience*.

[15] Park J. W., Vahidi B., Taylor A. M., Rhee S. W., Jeon N. L. (2006). Microfluidic culture platform for neuroscience research. *Nature Protocols*.

[16] Westermann S., Weber K. (2003). Post-translational modifications regulate microtubule function. *Nature Reviews Molecular Cell Biology*.

[17] Reed N. A., Cai D., Blasius T. L. (2006). Microtubule acetylation promotes kinesin-1 binding and transport. *Current Biology*.

[18] d'Ydewalle C., Bogaert E., Van Den Bosch L. (2012). HDAC6 at the intersection of neuroprotection and neurodegeneration. *Traffic*.

[19] Simões-Pires C., Zwick V., Nurisso A., Schenker E., Carrupt P.-A., Cuendet M. (2013). HDAC6 as a target for neurodegenerative diseases: what makes it different from the other HDACs?. *Molecular Neurodegeneration*.

[20] Rui Y., Tiwari P., Xie Z., Zheng J. Q. (2006). Acute impairment of mitochondrial trafficking by *β*-amyloid peptides in hippocampal neurons. *Journal of Neuroscience*.

[21] Vossel K. A., Zhang K., Brodbeck J. (2010). Tau reduction prevents A*β*-induced defects in axonal transport. *Science*.

[22] De vos K. J., Chapman A. L., Tennant M. E. (2007). Familial amyotrophic lateral sclerosis-linked SOD1 mutants perturb fast axonal transport to reduce axonal mitochondria content. *Human Molecular Genetics*.

[23] Trushina E., Dyer R. B., Badger J. D. (2004). Mutant huntingtin impairs axonal trafficking in mammalian neurons in vivo and in vitro. *Molecular and Cellular Biology*.

[24] Orr A. L., Li S., Wang C.-E. (2008). N-terminal mutant huntingtin associates with mitochondria and impairs mitochondrial trafficking. *Journal of Neuroscience*.

