# Supplementary material for: HDAC6 Inhibitors Rescued the Defective Axonal Mitochondrial Movement in Motor Neurons Derived from the Induced Pluripotent Stem Cells of Peripheral Neuropathy Patients with HSPB1 Mutation
Source: Stem Cells Int. 2016 Dec 26;2016:9475981. doi: 10.1155/2016/9475981 (PMC5220520; doi:10.1155/2016/9475981)
Supplement: Supplementary file 1 — Supplementary Material contains PCR detection of Sendai viral genome in iPSCs, and neuromuscular junction staining. It also contains mitochondrial movement data of different two clones from the patients. For comparison, immunostaining of acetylated alpha-tubulin data in S135F-MNs and P182L-MNs with HDAC6 inhibitors are included. [file 9475981.f1.pdf]

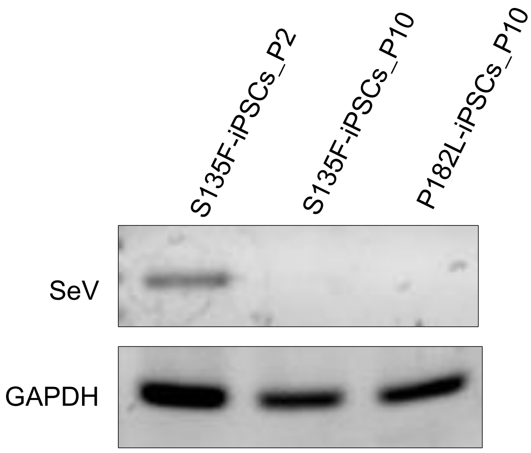

Supplement Figure S1. Assessment of Sendai viral genome contents in iPSCs. Sendai viral genome sequence (SeV) was expressed in S135F-iPSCs at cellular passages of 2 (P2) but SeV was not detected in S135F-iPSCs and P182L-iPSCs at cellular passages of 10 (P10). Primer sequences were 5'-GGA TCA CTA GGT GAT ATC GAG C-3' (forward) and 5'-ACC AGA CAA GAG TTT AAG AGA TAT GTA TC-3' (reverse) for SeV.

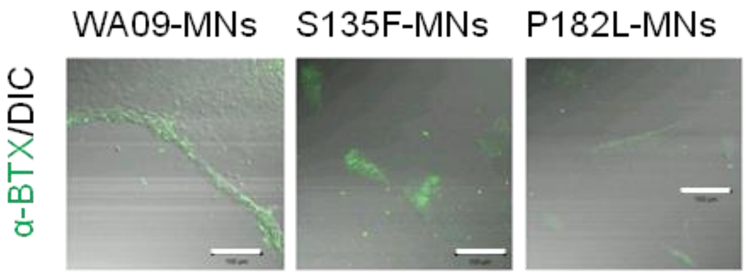

Supplement Figure S2. WA09-MNs, S135F-MNs and P182L-MNs formed neuromuscular junctions by co-culturing with myotube cells differentiated from C2C12 mice myoblast cells. Neuromuscular junctions were visualized by expression of acetylcholine receptors on myotube cells and staining with Alexa 488-conjugated α-BTX (DIC, differential interference contrast image; original magnification, 400x).

A

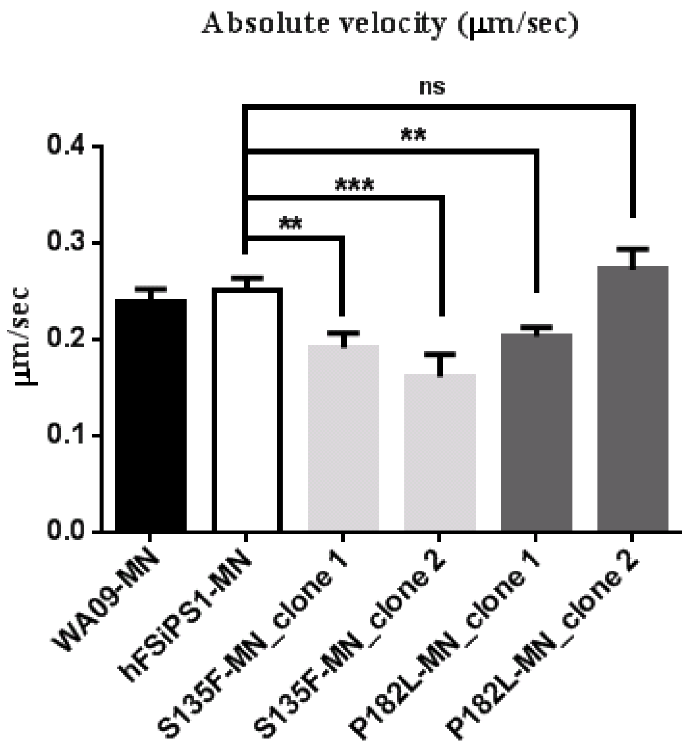

B

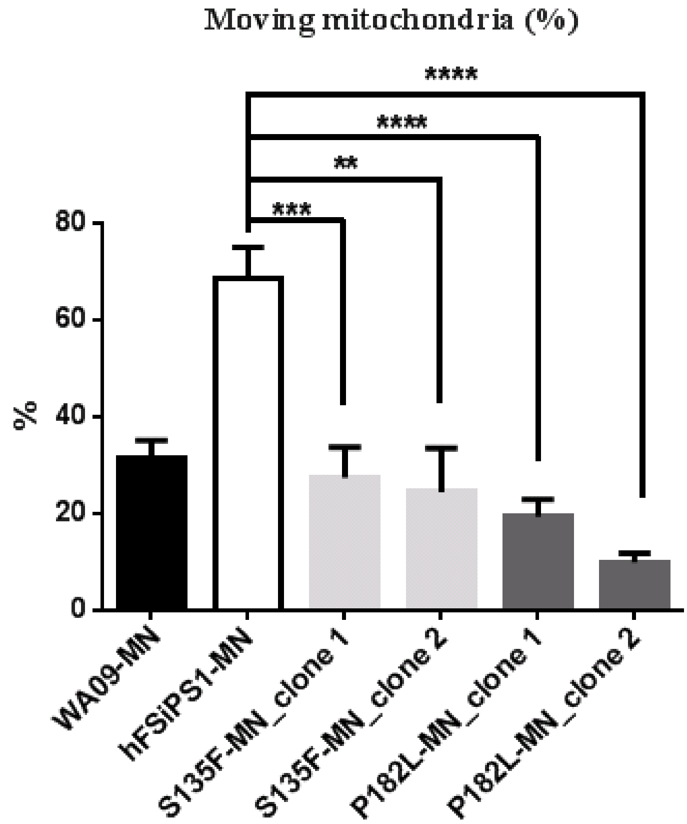

Supplement Figure S3 (Continued)

Supplement Figure S3. Axonal transport in S135F-MNs and P182L-MNs from two different iPSC clones. (**A**) Mitochondrial velocity in hFSiPS1-MNs (N=513), S135F-MNs_clone 1 (N=383), S135F-MNs_clone 2 (N=45), P182L-MNs_clone 1 (N=344) and P182L-MNs_clone 2 (N=152). (**B**) Moving mitochondria (%) in hFSiPS1-MNs (N=6), S135F-MNs_clone 1 (N=13), S135F-MNs_clone 2 (N=7), P182L-MNs_clone 1 (N=21), and P182L-MNs_clone 2 (N=21). Unpaired t-tests, *P<0.05, **P<0.01, ***P<0.001, and ****P<0.0001. ns, not significant.

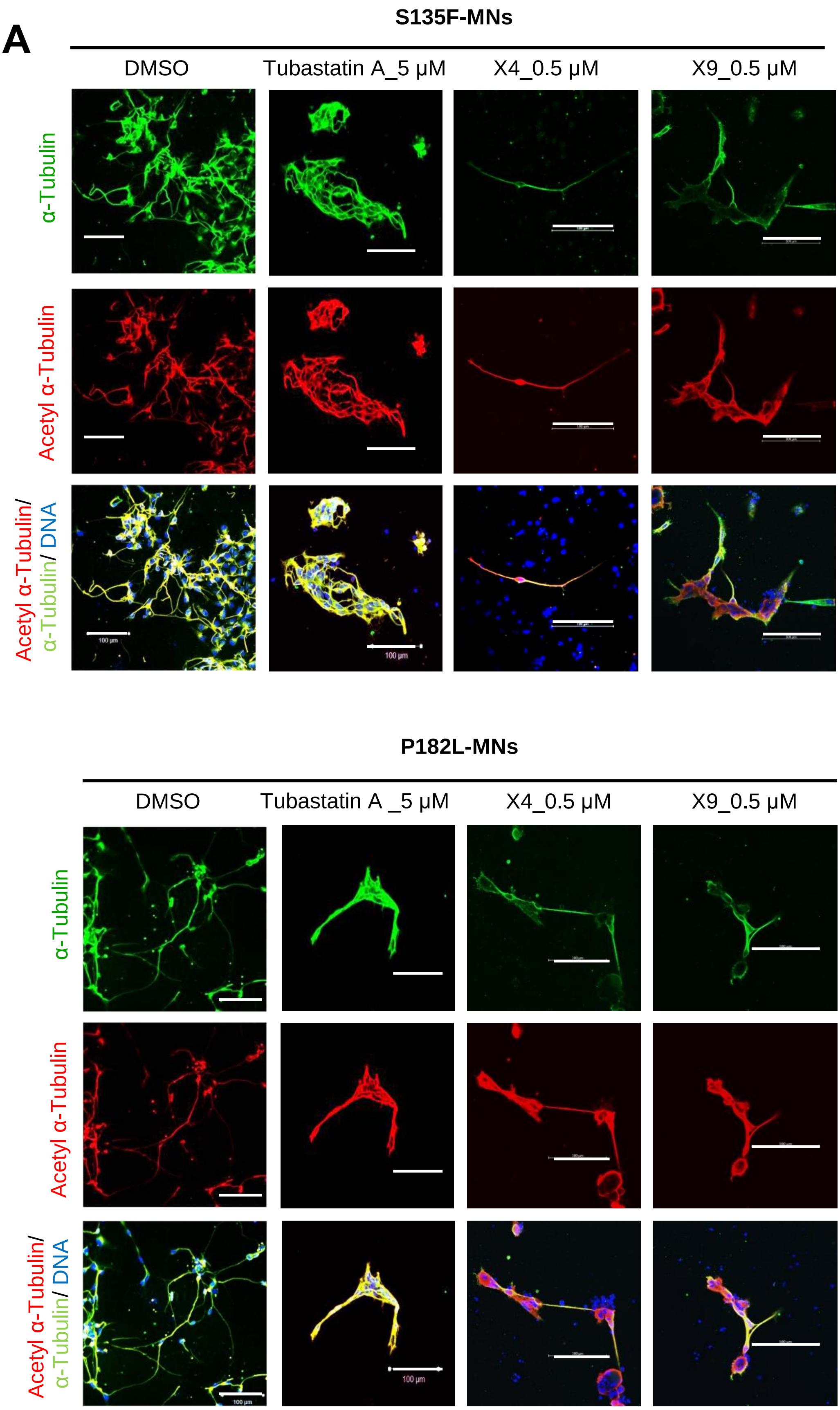

Supplement Figure S4 (Continued)

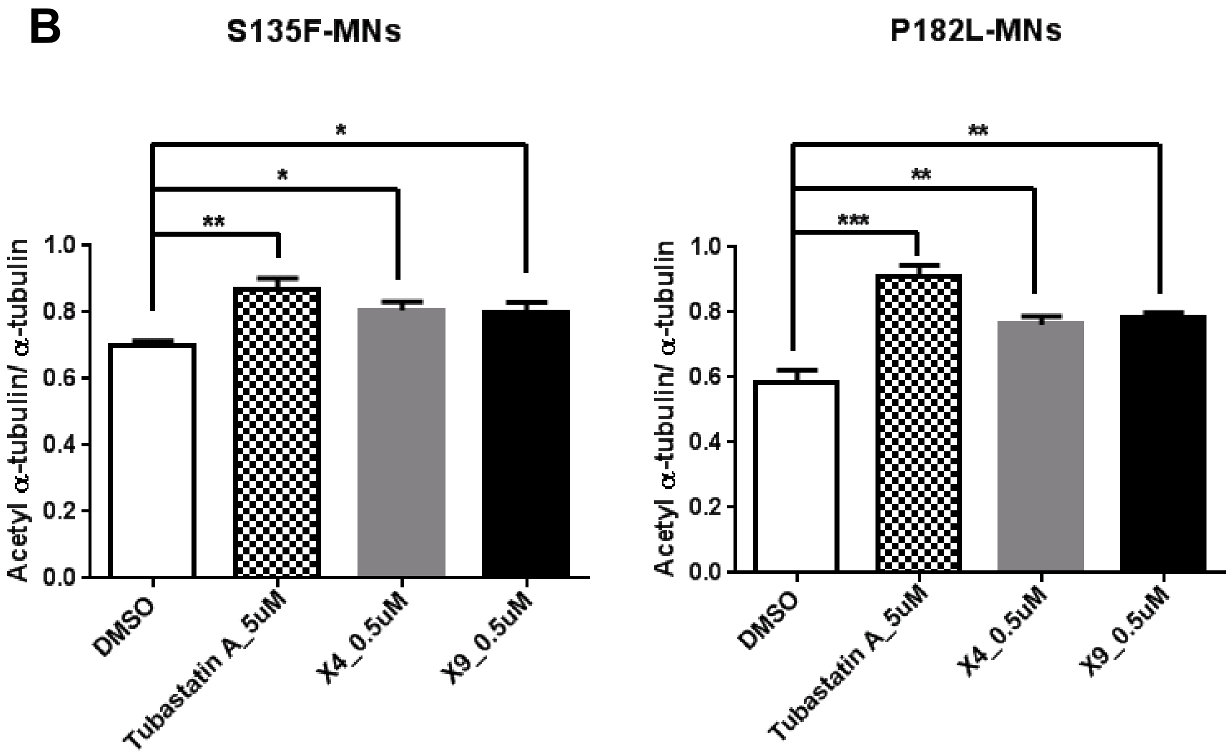

Supplement Figure S4. Immunostaining showed that treatment of HDAC6 inhibitors increased acetylated α-tubulin levels in S135F-MNs and P182L-MNs. **(A)** Acetylation of α-tubulin was visualized by immunostaining with anti-acetylated α-tubulin Abs (red). Total α-tubulin was also detected by staining with anti-α-tubulin Abs (green). (Original magnification, 400 ×). Scale bars: 100 μm. **(B)** The extent of acetylated α-tubulin was normalized with corresponding total α-tubulin using ImageJ software. Number of fields for image analysis were: S135F-MNs + DMSO (N=4), S135F-MNs + Tubastatin A (N=5), S135F-MNs + X4 (N=3), S135F-MNs + X9 (N=10), P182L-MNs + DMSO (N=5), P182L-MNs + Tubastatin A (N=4), P182L + X4 (N=4) and P182L-MNs + X9 (N=4). Unpaired t-tests, *P<0.05, **P<0.01 and ***P<0.001.

Supplement Table S1. Absolute velocity (μm/sec) of moving mitochondria

	DMSO	Tubastain A	X4		X9	
	0.01 %	5 μM	0.5 μM	5 μM	0.5 μM	5 μM
WA09-MNs	0.24 ± 0.01	0.26 ± 0.01	0.25 ± 0.01	0.33 ± 0.01	0.30 ± 0.01	0.30 ± 0.02
hFSiPS1-MNs	0.25 ± 0.01	-	-	-	-	-
S135F-MNs	0.19 ± 0.01	0.25 ± 0.02	0.48 ± 0.06	0.38 ± 0.03	0.25 ± 0.02	0.39 ± 0.02
P182L-MNs	0.22 ± 0.01	0.27 ± 0.02	0.28 ± 0.04	0.33 ± 0.02	0.23 ± 0.01	0.29 ± 0.01

Supplement Table S2. Percentage of moving mitochondria (%)

	DMSO	Tubastain A	X4		X9	
	0.01 %	5 μM	0.5 μM	5 μM	0.5 μM	5 μM
WA09-MNs	31.39 ± 3.74	49.71 ± 6.18	35.63 ± 6.28	49.74 ± 4.72	38.31 ± 6.02	34.07 ± 8.86
hFSiPS1-MNs	68.64 ± 6.42	-	-	-	-	-
S135F-MNs	26.37 ± 5.01	21.73 ± 6.53	58.40±10.42	48.31 ± 5.25	55.33 ± 5.86	62.88 ± 5.73
P182L-MNs	14.19 ± 2.14	30.91 ± 5.78	70.83 ± 4.17	37.59 ± 3.86	45.07 ± 4.48	33.14 ± 2.91

Supplement Materials and Methods

Neuromuscular junction formation

C2C12 mouse myoblast cells (CRL-1772, American Type Culture Collection (ATCC), Manassas, VA, USA) were cultured in DMEM supplemented with 10% FBS and 1 mM glutamine and penicillin/streptomycin. At a cell confluence of 70%, 1% insulin-transferrin-selenium supplement (Sigma) was added to the medium for myotube differentiation. After 2 days, 10 μM cytosine arabinoside (Sigma) was added to DMEM containing 0.5% FBS to remove dividing cells. After 2-4 days, differentiated myotubes were trypsinized and plated at a low density of 1×10^4 cells/well onto Matrigel-coated 8-well slide chambers (Nalgene/NUNC). After 1-2 days, CMT2F-MNs and ESC-MNs were co-cultured with myotubes at a ratio of 10:1 and supplemented with MN differentiation medium. After 1 week, functional neuromuscular junctions were visualized by staining MNs with Alexa 488-conjugated α-bungarotoxin (Invitrogen).
